# Supplementary material for: Translating genetics into tissue: inflammatory cytokine-producing TAMs and PD-L1 tumor expression as poor prognosis factors in cutaneous melanoma
Source: Front Immunol. 2025 May 8;16:1587545. doi: 10.3389/fimmu.2025.1587545 (PMC12095150; doi:10.3389/fimmu.2025.1587545)
Supplement: Supplementary file 1 [file DataSheet1.docx]

Supplementary Material

# Supplementary Data Figure Legends

**Supplementary Table S1.** List of Antibodies used in this study.

**Supplementary Table S2.** Association of evaluated markers with clinicopathological features (Mann–Whitney).

**Supplementary Table S3.** Gene sets used for DEG classification.

**Supplementary Figure S1. Melanoma associated macrophages phenotyping. (A)** Representative FFPE human primary melanoma and healthy lung samples stained for neutrophil CD66b (red) and CD15 (green) markers. Scale bar, 50 μm. **(B)** Number of both upregulated (red) and downregulated (blue) De of macrophages isolated from primary melanoma tumors and monocytes co-cultured with melanoma cells lines. **(C)** Classification of DEGs of primary tumor TAMs and monocytes co-cultured with melanoma cell lines according to gene sets proposed by Wei et al. **(D)** TAM cell average MFIs for SPP1, IL-4I1 and FLT1 in primary melanomas (arbitrary units, a.u.) and representative primary melanomas stained for TAM CD68^+^ (red) and SPP1, IL-4I1 and FLT1 (green). **(E)** Representative FFPE healthy colon and colon cancer samples stained for CD68 (macrophages, red) and FLT1 and HIF1A (green). **(F)** Single-TAM quantification of SPP1, CXCL9 and Activin A (antibody #2) showing three distinct expression patterns: dichotomic, widespread, and differently expressed between metastasizing conditions, respectively. **(G)** Dot-plots showing single-cell expression of Activin A (antibody #2) vs PD-L1, FOLR2, HIF1A and TNF in TAMs (n= 3 metastasizing primary melanomas). Spearman r >0.4 is considered biologically relevant.

**
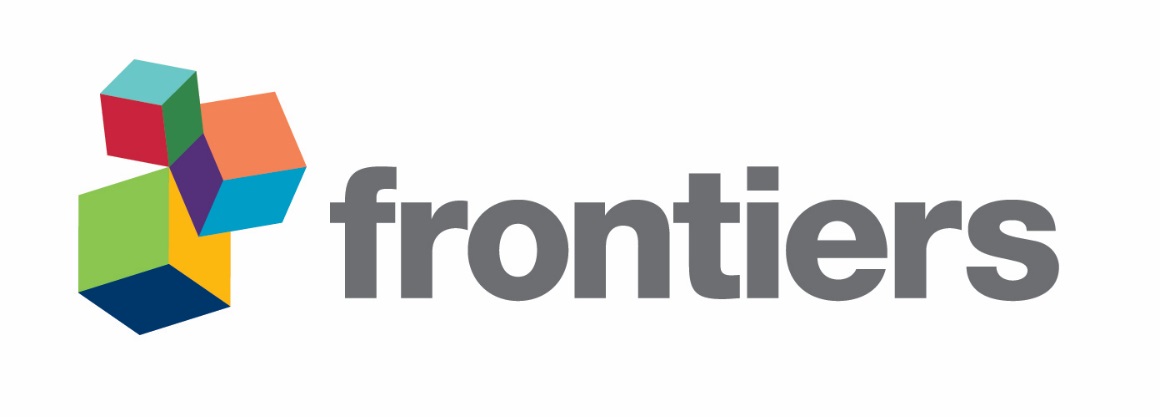
**
