# Supplementary figures and images for: Translating genetics into tissue: inflammatory cytokine-producing TAMs and PD-L1 tumor expression as poor prognosis factors in cutaneous melanoma
Source: Front Immunol. 2025 May 8;16:1587545. doi: 10.3389/fimmu.2025.1587545 (PMC12095150; doi:10.3389/fimmu.2025.1587545)

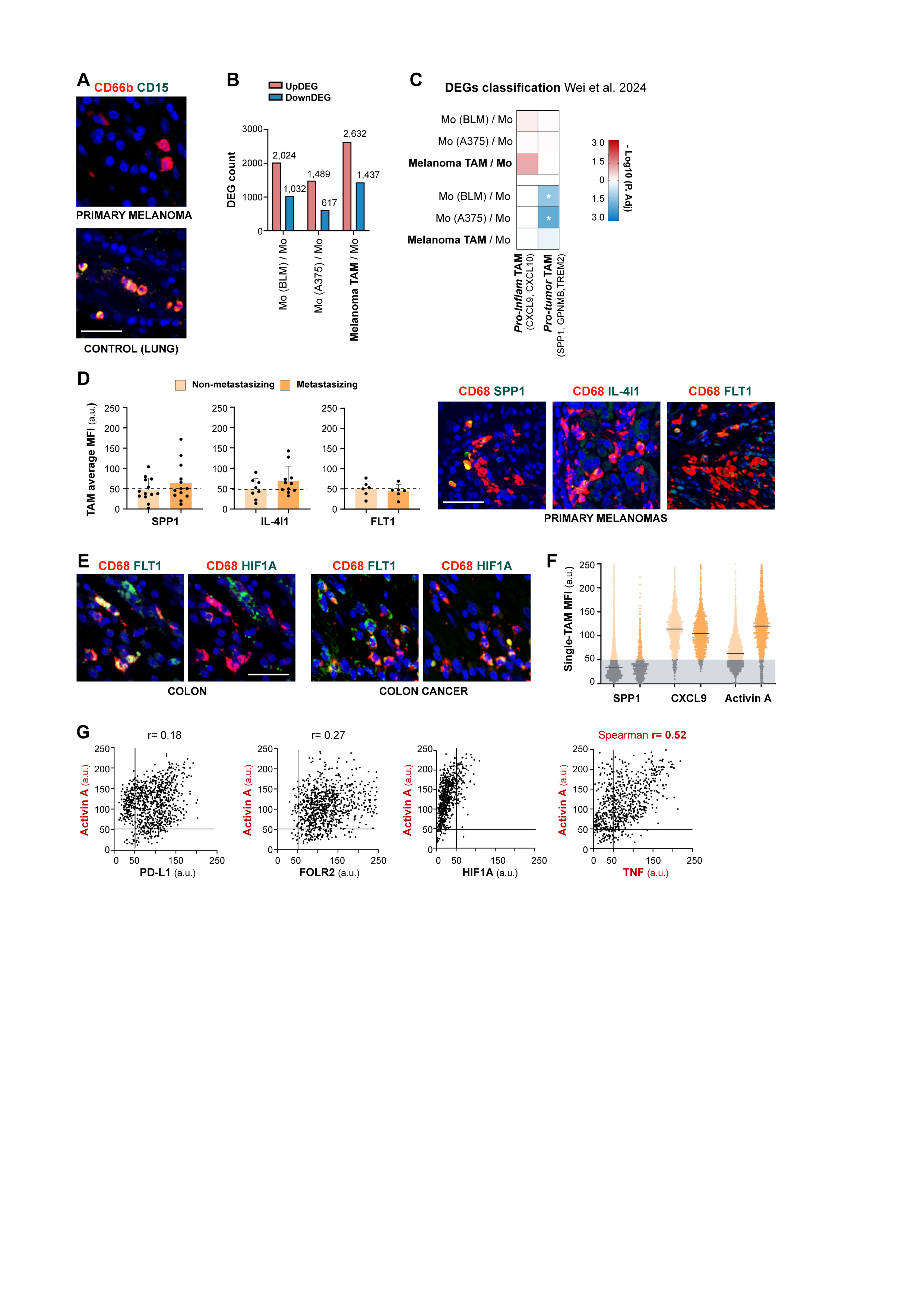

Supplement: Supplementary file 2 [file Image1.jpeg]
